# Atmospheric footprint of the recent warming slowdown

**DOI:** 10.1038/srep40947

**Published:** 2017-01-13

**Authors:** Bo Liu, Tianjun Zhou

**Affiliations:** 1 LASG, Institute of Atmospheric Physics, Chinese Academy of Sciences, Beijing 100029, China; 2 University of Chinese Academy of Sciences, Beijing 100049, China

## Abstract

Growing body of literature has developed to detect the role of ocean heat uptake and transport in the recent warming slowdown between 1998–2013; however, the atmospheric footprint of the slowdown in dynamical and physical processes remains unclear. Here, we divided recent decades into the recent hiatus period and the preceding warming period (1983–1998) to investigate the atmospheric footprint. We use a process-resolving analysis method to quantify the contributions of different processes to the total temperature changes. We show that the increasing rate of global mean tropospheric temperature was also reduced during the hiatus period. The decomposed trends due to physical processes, including surface albedo, water vapour, cloud, surface turbulent fluxes and atmospheric dynamics, reversed the patterns between the two periods. The changes in atmospheric heat transport are coupled with changes in the surface latent heat flux across the lower troposphere (below approximately 800 hPa) and with cloud-related processes in the upper troposphere (above approximately 600 hPa) and were underpinned by strengthening/weakening Hadley Circulation and Walker Circulation during the warming/hiatus period. This dynamical coupling experienced a phase transition between the two periods, reminding us of the importance of understanding the atmospheric footprint, which constitutes an essential part of internal climate variability.

Compared with the warming trend of the past several decades, the rate of increase of annual average global mean surface temperature (GMST) has experienced a reduction between 1998–2013, known as the hiatus^1^,^2^. However, climate models designed to represent the physics and dynamics of the climate system project that GMST continued to rise in the early 2000s^3^. Dominant mechanisms proposed to understand the hiatus included the internal climate variability^4^,^5^,^6^,^7^,^8^,^9^,^10^ and ocean heat uptake and transport^11^,^12^,^13^,^14^,^15^; however, the differences in the atmospheric footprint of recent warming slowdown remains unclear in terms of the dynamical and physical processes. Here, we use a process-resolving method to estimate the relative contributions of different processes to the trends of global mean tropospheric temperature. We find that the mean annual trends of tropospheric temperature and their vertical structures show distinct features, underpinned by opposite trends of atmospheric large-scale circulation during the recent warming hiatus and the preceding warming period (1983–1998). This provides us with a new perspective for better understanding the coupling mechanism between the ocean and the atmosphere of the recent warming slowdown.

Although some recent investigations have questioned the statistical robustness of the recent hiatus^16^,^17^, related works have noted that these statistics benchmark the recent slowdown against a baseline period that includes the so-called ‘big hiatus’ from the 1950s to the early 1970s^2^,^8^. Consistent with the reduction in the rate of increase in the GMST, satellite-based observations of increasing temperature of the lower troposphere (TLT) also experienced a slowdown^18^. Based on ERA-Interim dataset^19^, we have calculated a time series of anomalies (Fig. 1a) in the global mean near surface temperature (i.e., temperature of the lowest atmospheric layer), global mean troposphere average temperature (from surface to 100 hPa), lower troposphere average temperature (from surface to 500 hPa) and upper troposphere average temperature (from 500 hPa to 100 hPa). The warming trends of the global mean atmospheric temperatures (hereafter referred to as GMTs) for the hiatus period (1998–2013) are clearly smaller than those of the preceding warming period (1983–1998), except for the lower troposphere averaged temperature in the ERA-Interim (Fig. 1b). During the two periods, the behaviours of the global mean atmospheric vertical temperatures are consistent with observed GMST calculated from HadCRUT^20^, although the amplitudes differed. Note that for reanalysis datasets such as the ERA-Interim, the evolution of the atmospheric temperature is not only forced by surface, it is also blending many observed datasets during the assimilation process, including satellite and aircraft data and radiosonde temperature^19^. Hence, the evolution of global mean atmospheric temperatures in the ERA-Interim can be regarded as, to some degree, independent of observed GMST. Therefore, the recent warming slowdown between 1998–2013 is a consistent phenomenon from the surface up through the entire troposphere.

## Results

**Figure 2:**
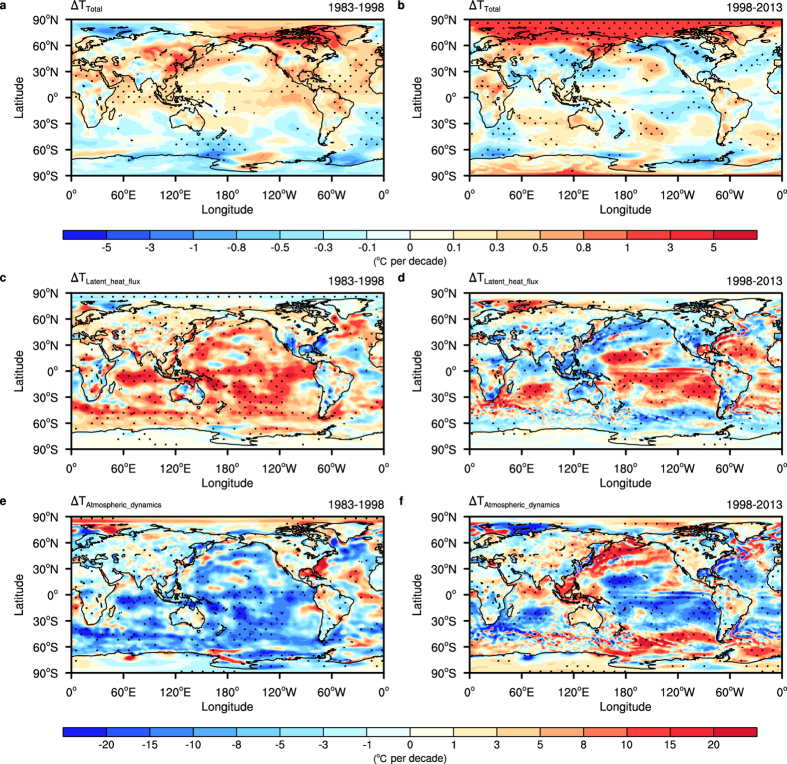
Near-surface temperature trends and their partial components due to latent heat flux and atmospheric dynamics for the hiatus period and the preceding warming period. Total near-surface temperature trends for (**a**) the warming period (1983–1998) and (**b**) the hiatus period (1998–2013) from the ERA-Interim dataset. Partial temperature trends due to (**c**,**d**) surface latent heat flux and (**e**,**f**) atmospheric dynamics for the two periods derived from CFRAM method. Trends statistically significant at the 5% level based on a Student’s *t*-test are dotted. This figure was created using NCAR Command Language (NCL) version 6.3.0 (http://www.ncl.ucar.edu/).

To determine the atmospheric footprint of the recent warming slowdown, we examine the horizontal and vertical distributions of the slowing down patterns. We first examined the observed trend distributions of GMTs during the two periods. Overall, these trends show large differences over the two periods – the near surface temperature shows warming trends over the tropical Pacific, North Atlantic, North Indian Ocean and Eurasia during the warming periods, while the trend is cooling across these regions during the hiatus period; from the near surface to the upper troposphere, the trend patterns become more uniform (Supplementary Fig. S1). Over the Pacific, the pattern of temperature trends resembles that of a positive IPO phase during the warming periods, with positive temperature trends over tropical regions and negative trends over adjacent extratropical regions; during the hiatus, a negative phase was found, which is consistent with previous studies that linked the recent warming hiatus to the negative IPO phase^4^,^7^. In addition, the pattern of Arctic temperature trends shows an apparent dipole structure with a warming/cooling trend over the eastern/western part during the warming period, while it demonstrates uniform warming during the hiatus period (Fig. 2; Supplementary Fig. S1).

To quantify the contributions of physical and dynamical processes to the observed temperature trends for the warming and hiatus periods, we calculate the trends of both periods and further use the climate feedback-response analysis method (CFRAM)^21^,^22^ to decompose the annual trends into partial components due to individual processes. The total trends bear a high similarity to the original near surface temperature trends (Fig. 2a,b and Supplementary Fig. S1a,b). To estimate the relative contributions from different processes to the total trends, we calculated the pattern amplitude projection coefficients (PAPs; Supplementary Fig. S2). The prominent contributions are from the surface latent heat flux and atmospheric dynamics (Supplementary Fig. S2), and the partial temperature trends due to these contributors show distinct differences between the two periods (Fig. 2 and Supplementary Fig. S3). For the preceding warming period, the surface latent heat flux contributes the most to the total warming trend while the atmospheric dynamics tend to balance the warming effect. During the hiatus period, both the dominant effect of the surface latent heat flux and the compensating effect of the atmospheric dynamics have reduced substantially. Since the latent heat flux is the strongest flux in the air-sea heat exchange, we conclude that the global ocean has released more heat to the atmosphere during the warming period, while this increasing trend was reversed over the Western Pacific warming pool, mid-latitude Pacific Ocean, North Indian Ocean and Southern Ocean during the recent hiatus period. The reduction in the upward surface latent heat flux from the ocean during the hiatus period is in agreement with changes in ocean heat uptake and transport noted in previous studies^11^,^12^,^13^,^14^.

The atmospheric dynamics terms are calculated using a residual method and further validated by independently calculating the trends in column integrated atmospheric transport from the surface to the top of the atmosphere (TOA) and the atmospheric transport derived from the radiative fluxes at TOA and the surface. The consistency of the atmospheric heat transport patterns (Supplementary Fig. S4) were calculated by two methods and indicated the robustness of the atmospheric transport term in CFRAM diagnosis. The opposite partial temperature trends, due to the surface latent heat flux and atmospheric heat transport (Fig. 2), indicate the dynamical coupling between the atmosphere and the ocean, which is also observed in the interaction between the forced climate change and the internal climate variability^23^. Moreover, the trends that resulted from the water vapour, cloud-related processes, and surface sensible heat flux also show apparent differences for the two periods (Supplementary Fig. S3).

**Figure 3:**
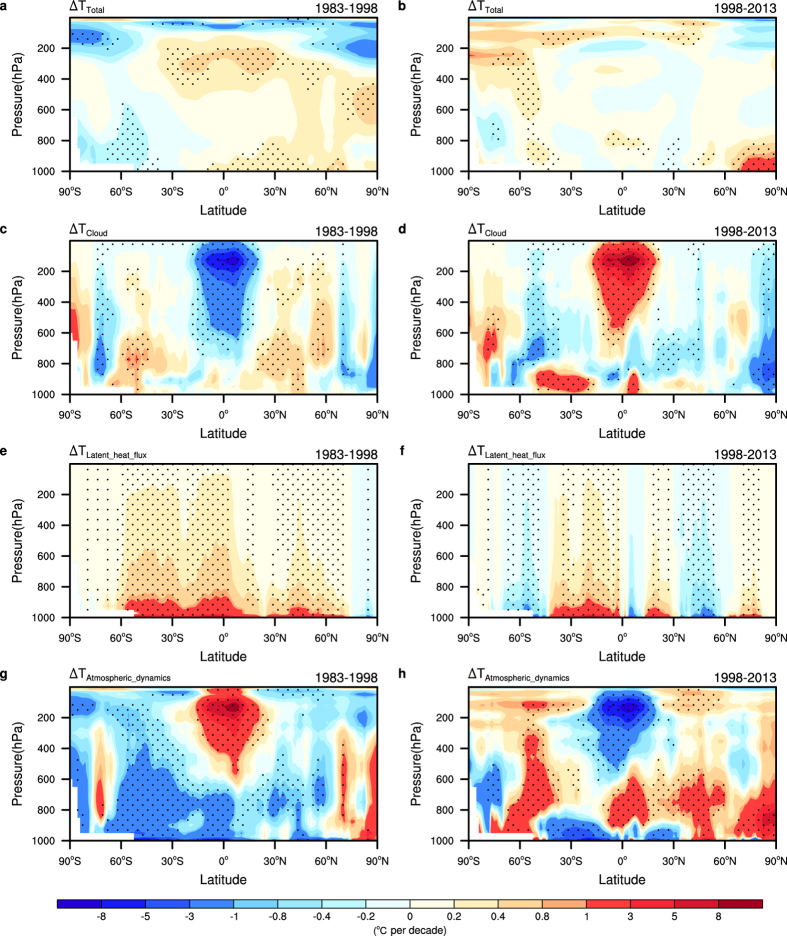
Temperature trends and their partial components due to cloud-related processes, surface latent heat flux and atmospheric dynamics for the hiatus period and the preceding warming period. Trends of zonal mean of annual mean temperature for (**a**) the warming period (1983–1998) and (**b**) the hiatus period (1998–2013) from the ERA-Interim dataset. Partial temperature trends due to (**c**,**d**) cloud-related processes, (**e**,**f**) surface latent heat flux and (**g**,**h**) atmospheric dynamics for the two periods derived from CFRAM method. Trends statistically significant at the 5% level based on a Student’s *t*-test are dotted.

**Figure 4:**
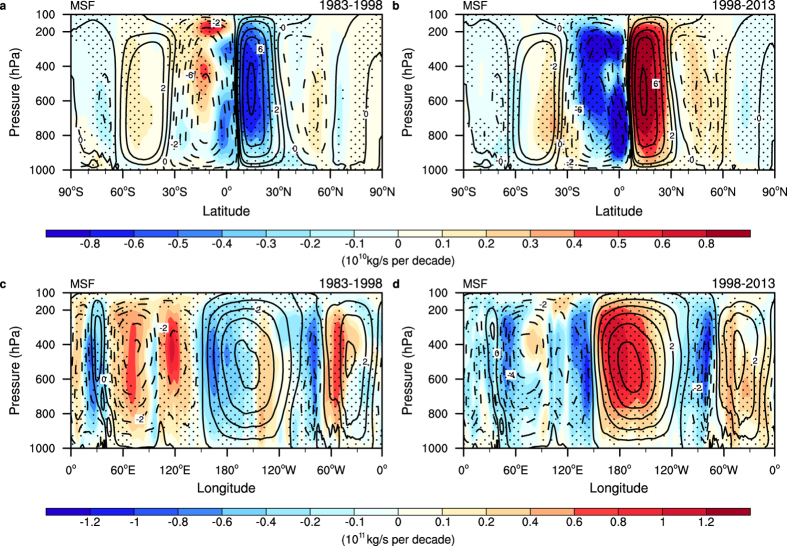
Linear trends of meridional and tropical zonal mass stream function (MSF) for the hiatus period and preceding warming period. Linear trends of the meridional MSF (shading) for (**a**) the warming period (1983–1998) and (**b**) the hiatus period (1998–2013) from ERA-Interim. Linear trends of tropical zonal MSF along the equator (5°S-5°N) (shading) for (**c**) the warming period and (**d**) the hiatus period from the ERA-Interim. Contours denote the multiyear mean of the meridional and tropical zonal MSF. Trends statistically significant at the 5% level based on a Student’s *t*-test are dotted.

To test whether the dynamical coupling mentioned above exists in the vertical structures of these trends, we have shown the decomposed results of total zonal mean temperature trends and their partial components for the two periods (Fig. 3; Supplementary Fig. S5). The vertical distribution of the total temperature trends for the two periods are evidently different; from the surface to approximately 200 hPa, the latitudes north of 30°S, overall, show an evident warming trend during the warming period; however, this trend has vanished during the hiatus period. Over the Arctic regions, the most salient feature is the middle-tropospheric (700 hPa–300 hPa) warming during the warming period in contrast to the lower-tropospheric (below 800 hPa) warming during the hiatus period. The lower-tropospheric warming during the hiatus period is dominated by atmospheric dynamics (Fig. 3), as manifested by anomalous southerly surface winds (Supplementary Fig. S6) and weakened atmospheric circulation over the polar region (Fig. 4), as observed in the regulation of IPO on Arctic amplification^24^. Due to the limitations of the surface latent heat flux in the lowest atmospheric layer, its contribution to the 3-dimensional temperature trend decreased significantly with increasing height and was mainly restricted to the lower troposphere. Moreover, the partial trends due to the surface latent heat flux and atmospheric transport tend to balance each other, indicating that the coupling between the atmospheric dynamics and the surface latent heat flux is confined to the lower troposphere (Fig. 3).

During the warming period, for the upper troposphere (above approximately 600 hPa), the atmospheric heat transport contributes the most to the total trend, while the cloud radiative effect (CRE) tends to compensate for it (Supplementary Fig. S2). Although both the globally averaged contributions of atmospheric transport and CRE are sharply reduced in terms of their absolute values (Supplementary Fig. S2), the trend distributions of both terms are opposite over the upper troposphere above the tropics (Fig. 3). Moreover, the zonal mean temperature trends due to water vapour and surface sensible heat flux also exhibit reversal trends between the two periods (Supplementary Fig. S5), as evidenced by the overall warming/cooling effect of water vapour over the warming/hiatus period. The opposite contributions of atmospheric dynamics and cloud suggest a dynamical coupling across the upper troposphere.

The tight coupling between the atmospheric dynamics and cloud-related processes indicates the importance of understanding the interaction between atmospheric circulation and clouds, which is crucial for large scale climate change^25^,^26^,^27^. We calculated the trends of meridional and tropical zonal mass stream function and the cloud fraction to illustrate this interaction over the two periods(see methods). During the warming period, the trend distribution of the near surface temperature resembles the pattern of temperature changes associated with the positive IPO phases. The deep convection over the Western Pacific Warming Pool (WPWP) is not fully developed, and convection over the Central Pacific is intensified, which manifests by a decreased high cloud fraction centred at approximately 150 hPa and an increased middle cloud fraction spreading from approximately 800 hPa to 300 hPa (Supplementary Fig. S7). Correspondingly, both the Walker Circulation (WC) and the Hadley Circulation (HC) have been weakening during this period. The weakened WC is also reflected by the reduced Pacific trade winds and anomalous sea level pressure (Supplementary Fig. S6), which is considered to be associated with the positive IPO phase, as documented in previous works^4^,^25^. Meanwhile, the poleward heat transport across the upper troposphere is reduced over the tropics (Supplementary Fig. S8). The weakening HC and decreasing poleward heat transport are associated with decreasing poleward temperature gradient due to cloud-related processes (Fig. 3c).

We have depicted how these atmospheric changes linked over the warming period. Next, we examine the atmospheric footprint of the hiatus period. The increased high cloud fraction above the WPWP implies that deep convection is fully developed, while the convective activity over the central Pacific is reduced (Supplementary Fig. S7). Accordingly, both the WC and the HC are strengthening, which is reflected by strengthened Pacific trade winds and increased poleward heat transport across the upper troposphere (Fig. 4b and Supplementary Fig. S8). To test whether the atmospheric footprint over the two periods is dominated by interannual or interdecadal variability, we regressed the IPO index^28^ on annual meridional and tropical zonal mass stream function derived from an atmospheric analysis dataset of 20^th^ century – ERA-20C^29^. Regression analysis indicated that the changes of the HC and the WC during the two periods were consistent with that of the IPO positive and negative phases (Fig. 4 and Supplementary Fig. S9).

By examining zonal mean cloud fraction trends, we find that centred at approximately 100 hPa in the upper troposphere over the tropics, the zonal mean cloud fraction is decreasing/increasing during the warming/hiatus period (Supplementary Fig. S10). Overall, the cloud radiative effect (CRE) is dominated by long wave CRE (LWCRE), while the shortwave CRE (SWCRE) tends to counteract it. Both trends of SWCRE and LWCRE contribute to the total CRE trend over tropical upper troposphere (Supplementary Fig. S10). The overall cooling/warming effect is associated with the warming/cooling effect of the atmospheric heat transport and, thus, is linked to the weakening/strengthening atmospheric circulation.

### Summary

We show evidence that trend of the slowdown in surface temperature warming is also evident in the troposphere. The slowdown is a phase transition of internal climate variability with an evident atmospheric footprint, including interactions between large-scale circulation and cloud distribution. New evidence shows that the global warming trend resumed in 2014^2^,^30^ because the IPO transitioned from negative to positive^30^, and 2014 and 2015 are now the warmest two years on record^2^,^8^. Future projections of climate need a better understanding of the combined effects of both external forcings and internal variability. Regardless of the variability of the external forcings, we believe that there is a probability that the atmospheric footprint of future decades resembles that of the preceding warming period (1983–1998), although the time span may depend on this ongoing positive IPO phase.

## Methods

### Observation and Reanalysis Data

The observed surface temperature used in this study is from the Hadley Centre and the Climate Research Unit combined land SAT and SST (HadCRUT) version 4.3.0.0 (http://www.metoffice.gov.uk/hadobs/crutem4/)^20^. The other variables, including 3D variables – air temperature, meridional wind, zonal wind, relative humidity, cloud fraction, cloud liquid/ice water content – and 2D variables – surface albedo, surface sensible and latent heat flux, TOA incident solar radiation, sea level pressure, 10 m winds – are obtained from the interim European Centre for Medium-Range Weather Forecasts (ECMWF) Reanalysis (ERA-Interim) dataset^19^. We also used meridional wind, zonal wind, surface pressure from ECMWF’s first atmospheric reanalysis of the 20^th^ century (ERA-20C)^29^.

### Trend Decomposition Analysis

Given the time span of the recent hiatus period (1998–2013), we have chosen the preceding 16 years (1983–1998) as a contrast to examine the atmospheric footprint of the recent hiatus period. The CFRAM has been adopted to quantify contributions of various processes in the surface and atmospheric temperature anomalies associated with El Niño-Southern Oscillation (ENSO)^31^,^32^, and the Northern Annular Mode (NAM)^33^. Also, this framework is used to analyze the temperature bias in climate models^34^,^35^,^36^. Here, we used CFRAM to estimate the contributions of individual processes to the total temperature trends over the two periods. CFRAM provides the possibility to decompose the total observed temperature changes into partial components due to various processes^21^,^22^; these partial components are additive, and their sum is equal to the total change. See details in Supplementary Information.

### Meridional and Tropical Zonal Mass Streamfunction

The meridional mass streamfunction is computed by vertically integrating the zonal mean density-weighted meridional wind from TOA downward^37^. The tropical zonal mass streamfunction is computed by vertically integrating density-weighted divergent component of zonal wind averaged over 5°S-5°N from TOA downward^38^.

## Additional Information

**How to cite this article**: Liu, B. and Zhou, T. Atmospheric footprint of the recent warming slowdown. *Sci. Rep.* **7**, 40947; doi: 10.1038/srep40947 (2017).

**Publisher's note:** Springer Nature remains neutral with regard to jurisdictional claims in published maps and institutional affiliations.

## Supplementary Material

Supplementary Information

## Figures and Tables

**Figure 1:**
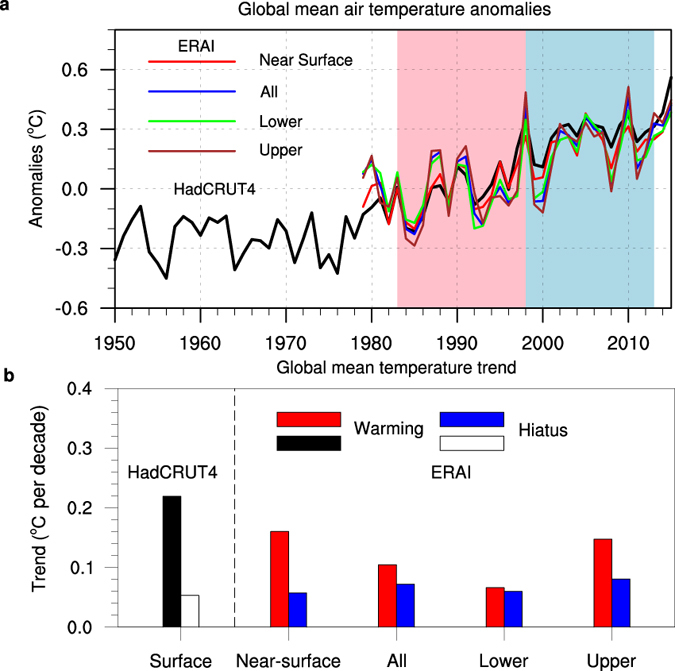
Time series of global mean temperature anomalies from 1950 to 2010 and trends of global mean temperature for near-surface, and the whole, lower, and upper troposphere. (**a**) Time series of annual mean of anomalies in global mean surface temperature derived from the HadCRUT4 dataset, and global mean temperature anomalies derived from ERA-Interim dataset for near surface (red), and vertical average of the whole troposphere (blue; from the surface to 100 hPa), lower troposphere (green; from the surface to 500 hPa) and upper troposphere (brown; from 500 hPa to 100 hPa). (**b**) Global mean surface temperature trend from the HadCRUT4 dataset, the global mean temperature trends for near surface, and vertical average of the whole troposphere, lower troposphere and upper troposphere for the hiatus period (1998–2013) and preceding warming period (1983–1998).
