# Supplementary material for: Atmospheric footprint of the recent warming slowdown
Source: Sci Rep. 2017 Jan 13;7:40947. doi: 10.1038/srep40947 (PMC5233970; doi:10.1038/srep40947)
Supplement: Supplementary Information [file srep40947-s1.pdf]

Supplementary information

Atmospheric footprint of recent warming slowdown

Bo Liu, Tianjun Zhou

Brief Introduction of CFRAM Formulation

The CFRAM[1,2] is based on the total energy balance within the multilayer surface-atmosphere column at a specific horizontal grid with one, two, or three dimensions. For a specific horizontal grid, the total energy balance can be written as

$$\vec{R} = \vec{S} + \vec{Q}^{nonradiative} - \frac{\partial \vec{E}}{\partial t}, \tag{1}$$

indicating that within each atmospheric layer the divergence of longwave radiation flux ($\vec{R}$, W/m^2) is always equal to the sum of the convergence of shortwave radiation flux ($\vec{S}$), total energy influx due to non-radiative processes ($\vec{Q}^{nonradiative}$), and the decrease of heat storage in the column ($-\frac{\partial \vec{E}}{\partial t}$). Due to the small heat capacity of the air, the heat storage term is close to zero even on a seasonal time-scale. Therefore, when considering the difference between two time mean states, equation (1) can be written as

$$\Delta\vec{S} - \Delta\vec{R} + \Delta\vec{Q}^{nonradiative} = 0 , \tag{2}$$

According to the linear approximation for the perturbation of radiative transfer in CFRAM, the interactions among various radiative feedback processes can be assumed to be approximately negligible, and, thus, the perturbed longwave cooling and convergence of shortwave heating can be linearized as follows:

$$\Delta\vec{S} \approx \Delta\vec{S}^{(\alpha)} + \Delta\vec{S}^{(w)} + \Delta\vec{S}^{(c)} , \tag{3}$$

$$\Delta\vec{R} \approx \Delta\vec{R}^{(\mathrm{w})} + \Delta\vec{R}^{(\mathrm{c})} + \frac{\partial\vec{R}}{\partial\vec{T}}\Delta\vec{T}, \tag{4}$$

where superscripts α, w, and c represent surface albedo, water vapour, and cloud, respectively. $\Delta\vec{T}$ is the temperature difference between the two climate states. $\frac{\partial\vec{R}}{\partial\vec{T}}$ is the Planck feedback matrix[17]. The energy perturbation associated with non-radiative processes can be further divided into energy perturbations related to surface sensible/latent heat flux, L/OHT and atmospheric dynamics:

$$\Delta\vec{Q}^{nonradiative} = \Delta\vec{Q}^{(\mathrm{SH})} + \Delta\vec{Q}^{(\mathrm{LH})} + \Delta\vec{Q}^{(\mathrm{atmos\_dyn})}, \tag{5}$$

In equation (5), $\Delta\vec{Q}^{(\mathrm{SH})}$ and $\Delta\vec{Q}^{(\mathrm{LH})}$ are energy differences due to changes in surface sensible/latent heat flux, and both of them have zero values in all except the lowest atmospheric layer. $\Delta\vec{Q}^{(\mathrm{atmos\_dyn})}$ stands for energy differences due to turbulent, convective and large-scale atmospheric motions.

Substituting equations (3-5) into equation (2), and multiplying both sides of the resultant equation by $\left(\frac{\partial\vec{R}}{\partial\vec{T}}\right)^{-1}$, and then rearranging these terms, we obtain

$$\begin{aligned}\Delta\vec{T} = \left(\frac{\partial\vec{R}}{\partial\vec{T}}\right)^{-1} \Big\{ & \Delta\vec{S}^{(\alpha)} + \Delta\left(\vec{S} - \vec{R}\right)^{(\mathrm{w})} + \Delta\left(\vec{S} - \vec{R}\right)^{(\mathrm{c})} \\ & + \Delta\vec{Q}^{(\mathrm{SH})} + \Delta\vec{Q}^{(\mathrm{LH})} + \Delta\vec{Q}^{(\mathrm{atmos\_dyn})} \Big\}\end{aligned}, \tag{6}$$

By applying Equation (6) we may decompose the local surface temperature anomalies ($\Delta\vec{T}$) into seven partial temperature changes due to (left to right) surface albedo, water vapour, cloud, surface sensible/latent heat flux, and atmospheric dynamics.

Trend Decomposition

Here, to minimize the effect of interannual variability on calculations of temperature

difference, CFRAM has been applied to obtain partial temperature differences of every single year with respect to the whole period mean, to calculate the total temperature trends and their partial components. The details are as follows:

1) Obtain the decomposed temperature differences of every single year, with respect to the whole period mean, for the six processes listed above. In particular, for the preceding warming period,

$$\Delta T_{year} = \sum_{i=1}^{6} \Delta T_i \quad \text{(year = 1983, 1984, …, 1997, 1998)}, \tag{7}$$

where ΔT_{year} is the total temperature anomalies of a single year relative to the mean temperature of the accelerated warming period (1983-1998), and ΔT_i is the partial temperature anomalies derived from CFRAM. Similarly, for the recent warming hiatus period,

$$\Delta T_{year} = \sum_{i=1}^{6} \Delta T_i \quad \text{(year = 1998, 1999, …, 2012, 2013)}. \tag{8}$$

2) Calculate the trends of ΔT_{year} and corresponding ΔT_i for both periods. According to previous works[17,18], the partial temperature differences, as derived from CFRAM, are additive and the sum should be approximately equal to the total temperature difference[17,18]. For the preceding warming period, we obtain

$$(\Delta T_{year})' = \sum_{i=1}^{6} (\Delta T_{i,year})' \quad \text{(year = 1983, 1984, …, 1997, 1998)}, \tag{9}$$

i.e., the temperature trends can be decomposed into partial components due to different processes and their sum should be equal to the original total trend. The same applies to the recent warming hiatus period.

Pattern amplitude projection

To quantify the relative contributions of the six processes to the amplitude of temperature changes over certain regions, we calculated pattern-amplitude projection (PAP) coefficients[28,29]. The PAP coefficient is defined as

$$PAP_i = A^{-1}\int_A a^2 \Delta T \cos\phi d\lambda d\phi \cdot \frac{A^{-1}\int_A a^2 \Delta T_i \Delta T \cos\phi d\lambda d\phi}{A^{-1}\int_A a^2 \Delta T^2 \cos\phi d\lambda d\phi}, \quad (7)$$

where ϕ and λ are latitude and longitude, respectively; a is the mean radius of the earth, and A is the area of the region under consideration. ΔT and ΔT_i are the total temperature change and partial temperature changes associated with individual processes for a certain grid-point. By definition, PAP is the weighted area average of the total temperature bias multiplied by a "pattern projection coefficient". Since the sum of the pattern projection coefficients equals 1, the sum of all the PAPs due to these processes for a certain region equals exactly the area average of the observed temperature change. In this sense, PAP_i can provide a more reasonable measure when assessing the relative contributions of individual processes to the observed total temperature changes.

References

1. Lu, J. & Cai, M. A new framework for isolating individual feedback processes in coupled general circulation climate models. Part I: Formulation. *Clim. Dyn.*, **32,** 873–885 (2009).
2. Cai, M. & Lu, J. A new framework for isolating individual feedback processes in coupled general circulation climate models. Part II: Method demonstrations and

comparisons. *Clim. Dyn.* **32,** 887–900 (2009).

3. Park, T. W., Deng, Y. & Cai, M. Feedback attribution of the El Niño–Southern Oscillation–related atmospheric and surface temperature anomalies, *J. Geophys. Res.* **117,** D23101(2012).

4. Park, T. W., Deng, Y., Cai, M., Jeong, J. H. & Zhou, R. A dissection of the surface temperature biases in the community earth system model. *Clim. Dyn.* **43,** 2043–2059 (2014).

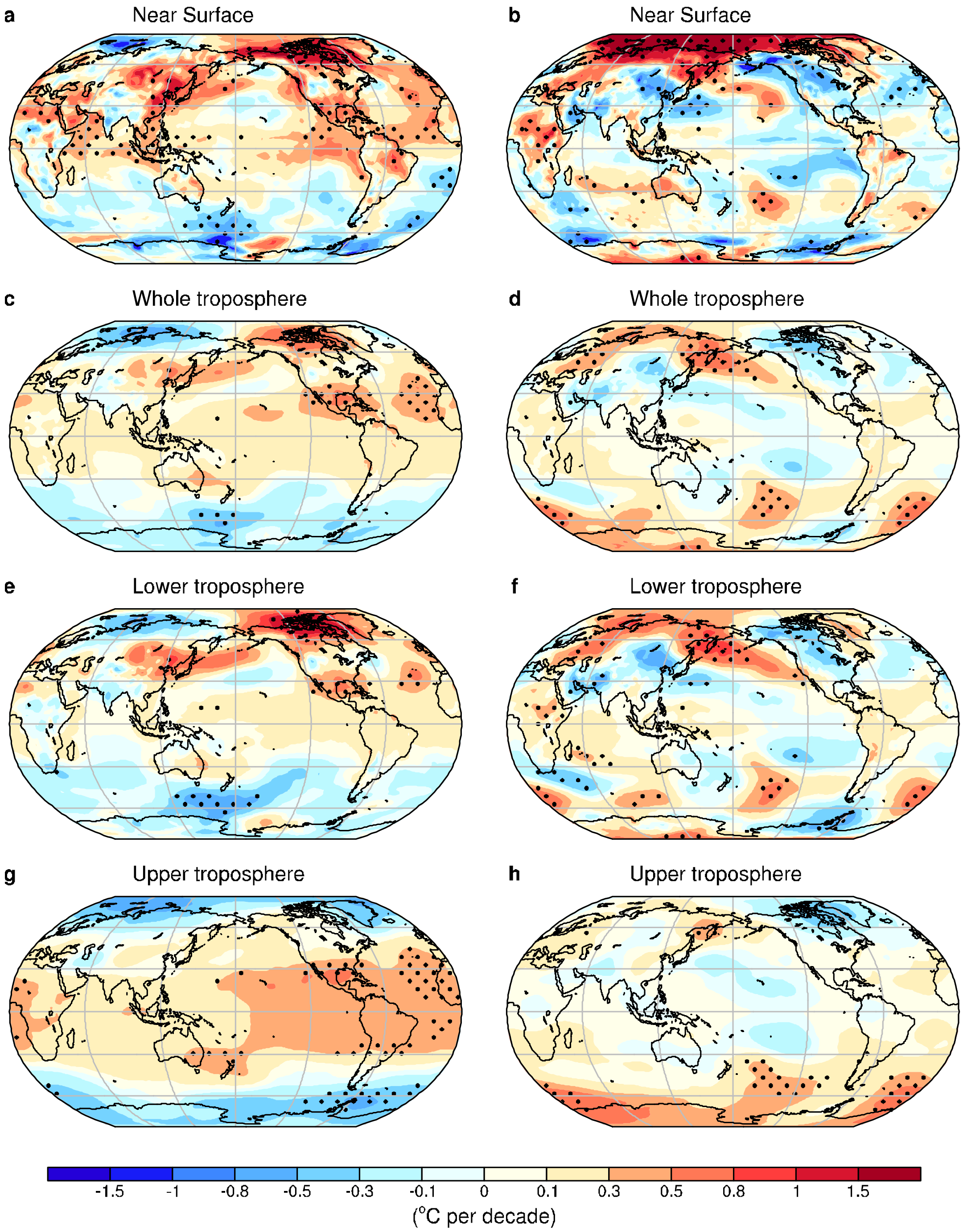

Fig. S1. Distribution of atmospheric temperature trends. Trends of (**a, b**) near surface (i.e. the lowest atmospheric layer), (**c, d**) whole troposphere (from surface to 100hPa), (**e, f**) lower troposphere (from surface to 500hPa) and (**g, h**) upper troposphere (from 500hPa to 100hPa) average temperature for (left) the warming period (1983-1998) and (right) the hiatus period (1998-2013) derived from ERA-Interim. Trends statistically significant at the 5% level based on a Student's *t*-test are dotted. This figure was created using NCAR Command Language (NCL) version 6.3.0 (http://www.ncl.ucar.edu/).

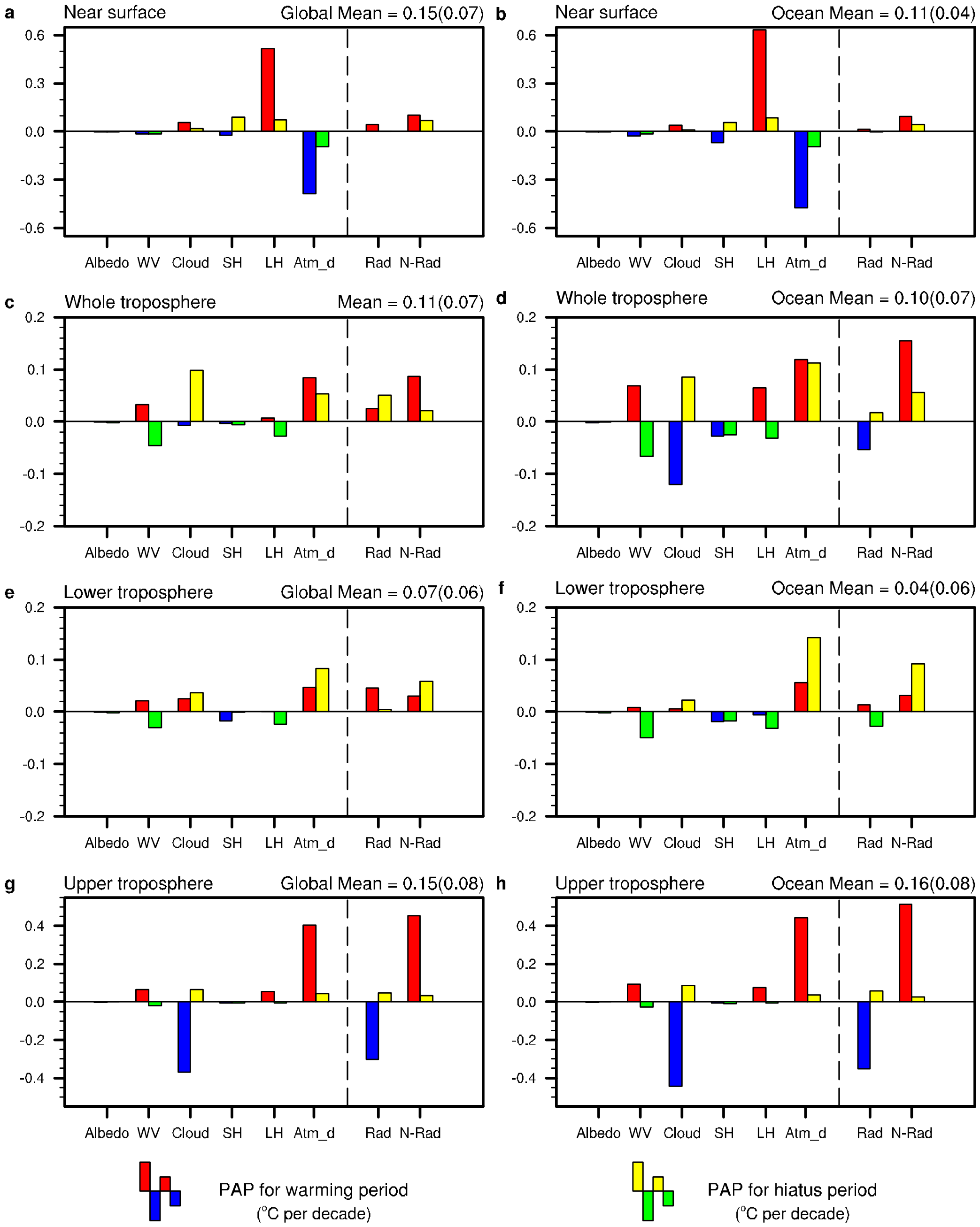

Fig. S2. Pattern-Amplitude Projection (PAP) Coefficients of the atmospheric temperature trends and their partial components. PAP of temperature trends and their partial components of (**a, b**) lowest atmospheric layer, (**c, d**) whole troposphere (from surface to 100hPa), (**e, f**) lower troposphere (from surface to 500hPa), (**g, h**) upper troposphere (from 500hPa to 100hPa) for (left) globe and (right) ocean for the warming period and the hiatus period.

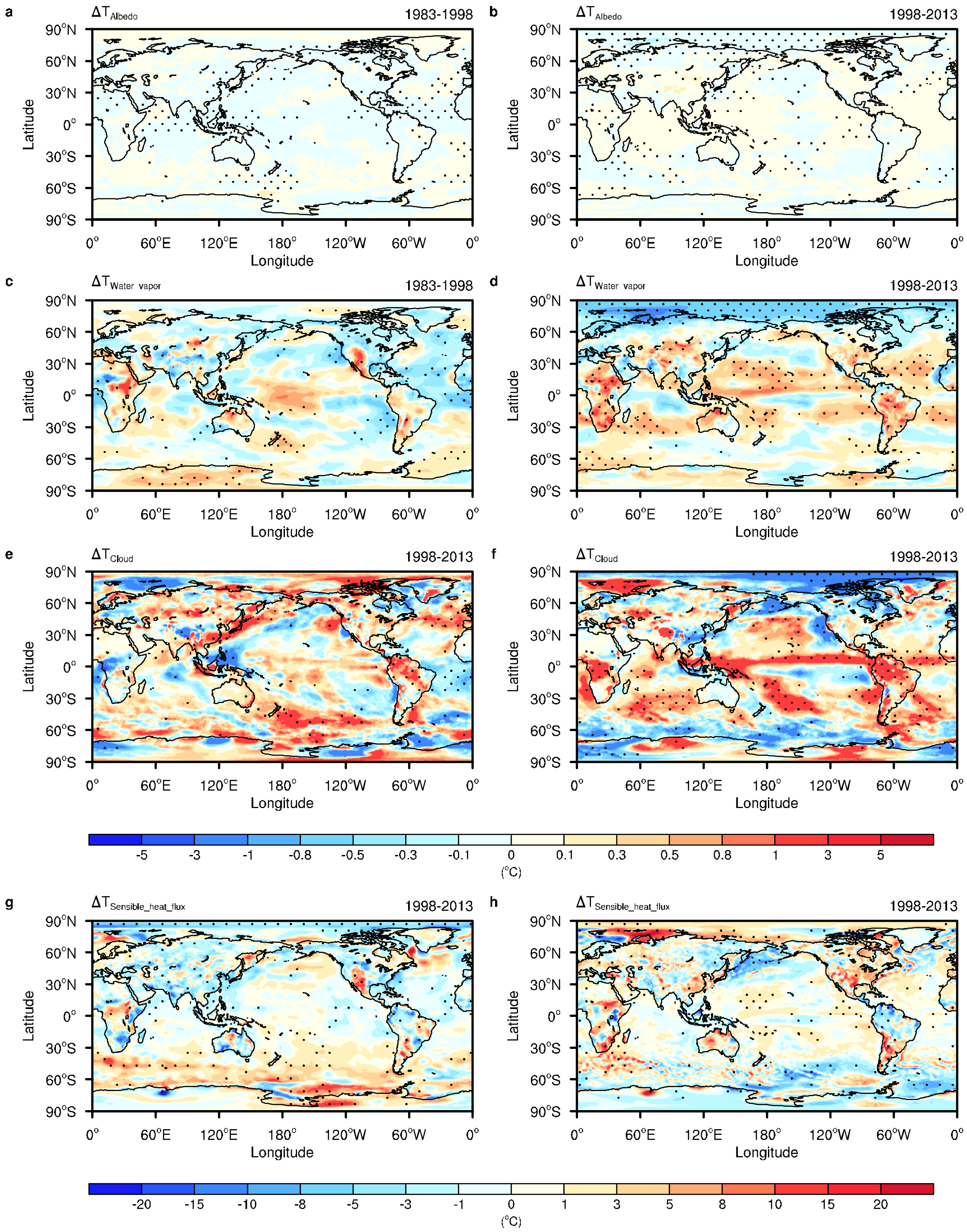

Fig. S3. Partial temperature trends of near surface. Partial temperature trends due to (**a, b**) surface albedo, (**c, d**) water vapor, (**e, f**) cloud-related processes and (**g, h**) surface sensible heat flux for (left) the warming period and (right) the hiatus period, derived from CFRAM method. Trends statistically significant at the 5% level based on a Student's *t*-test are dotted. This figure was created using NCAR Command Language (NCL) version 6.3.0 (http://www.ncl.ucar.edu/).

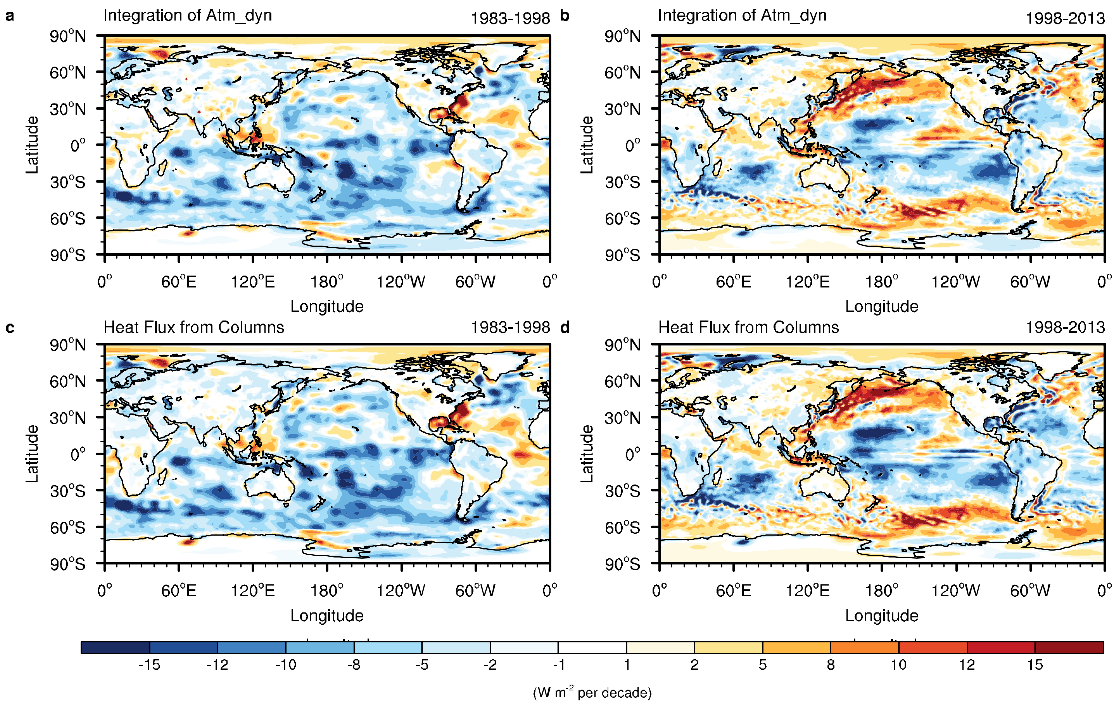

Fig. S4. Validation of calculation of atmospheric dynamics terms. Trends of tropospheric atmospheric dynamics derived from (**a, b**) integration of atmospheric dynamics terms from surface to the TOA, and (**c, d**) the difference between radiative fluxes at TOA and at the surface for (left) the warming period and (right) the hiatus period. This figure was created using NCAR Command Language (NCL) version 6.3.0 (http://www.ncl.ucar.edu/).

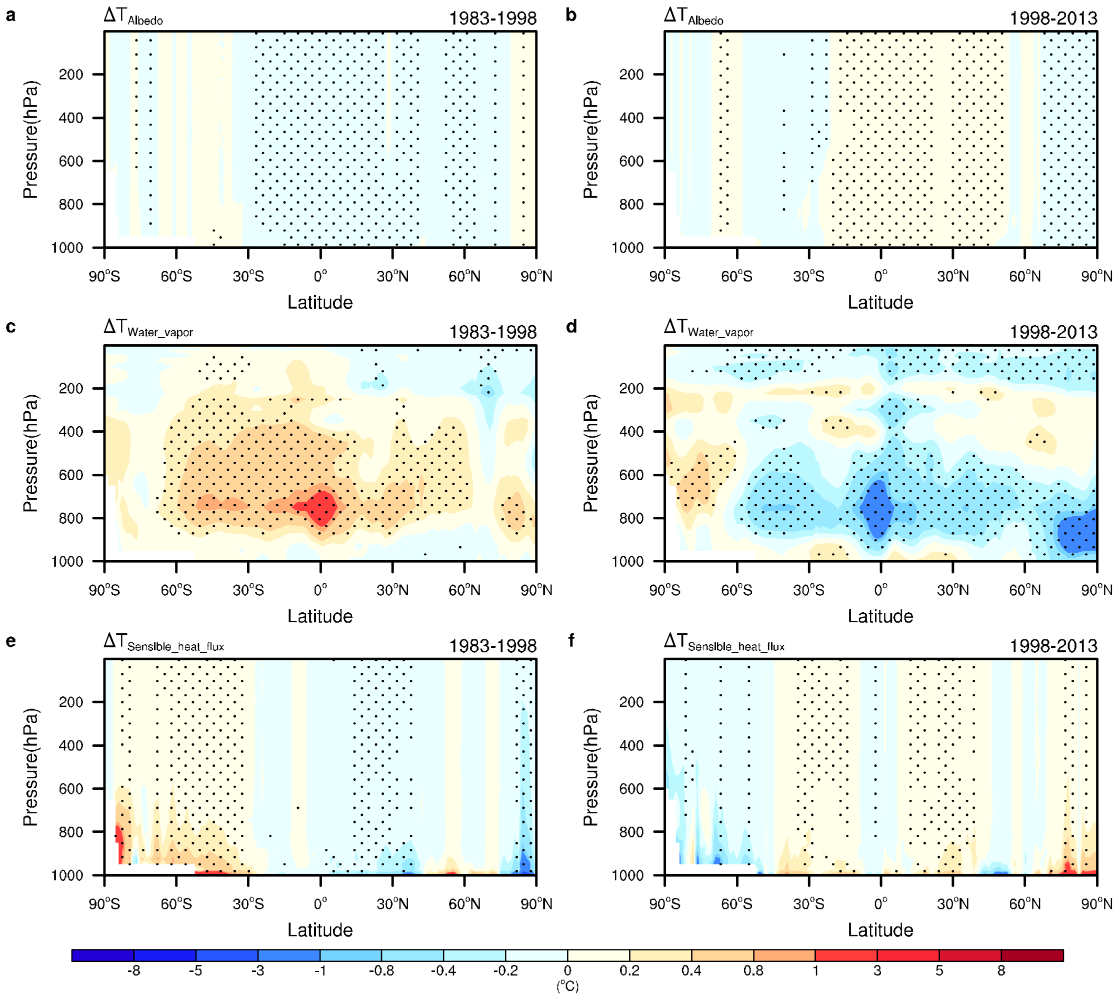

Fig. S5. Partial temperature trends. Zonal Average partial temperature trends due to surface albedo (**a, b**), water vapor (**c, d**) and atmospheric dynamics (**e, f**) for the two periods derived from CFRAM method. Trends statistically significant at the 5% level based on a Student's *t*-test are dotted.

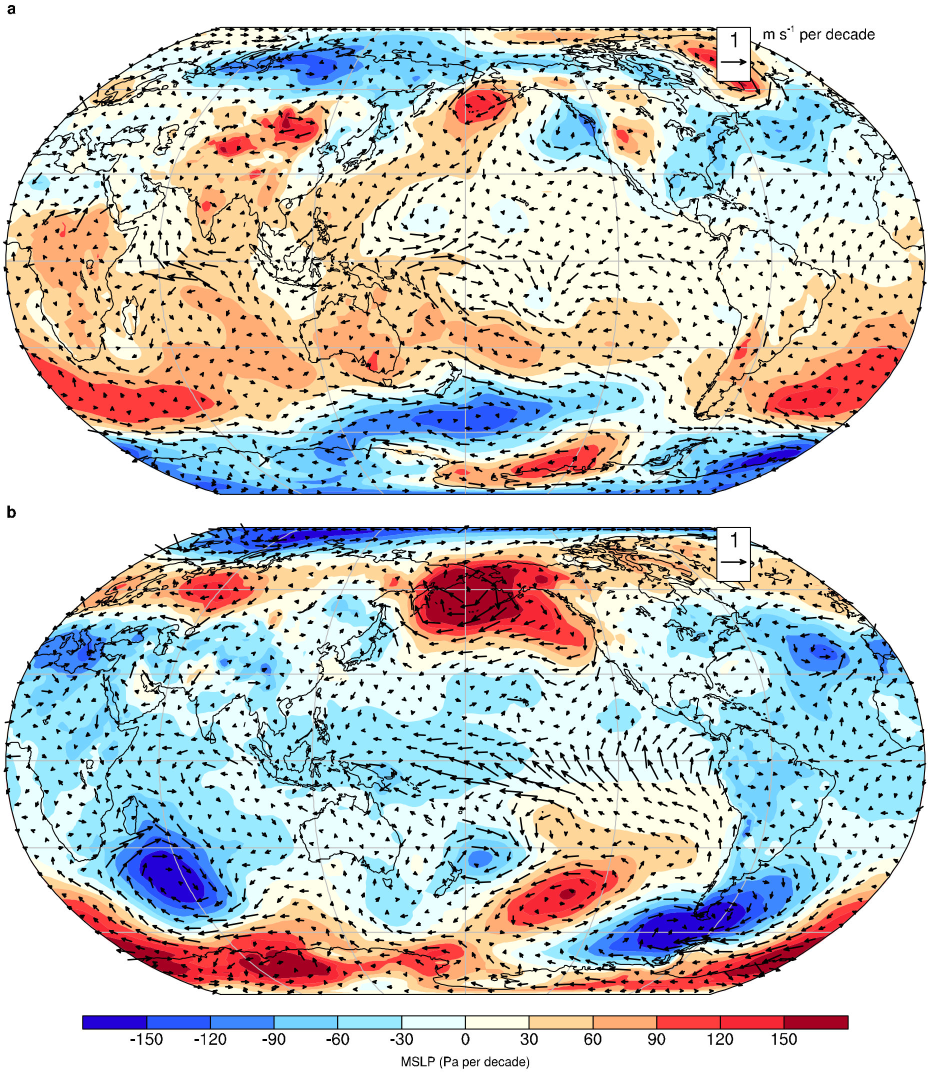

Fig. S6. Trends of annual mean sea level pressure (MSLP) and surface wind vectors. Trends of MSLP and surface wind vectors (at 10m) for (**a**) the warming period and (**b**) the hiatus period from ERA-Interim. This figure was created using NCAR Command Language (NCL) version 6.3.0 (http://www.ncl.ucar.edu/).

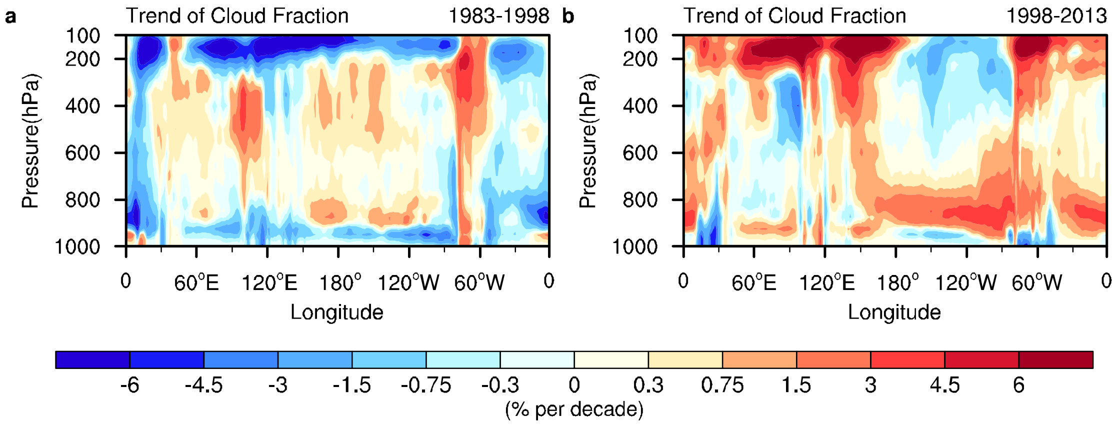

Fig. S7. Trends of meridional mean of annual mean cloud fraction over tropics for the warming and the hiatus period. Trends of meridional mean of annual mean cloud fraction along the equator (5°S-5°N) for (**a**) the warming period and (**b**) the preceding hiatus period.

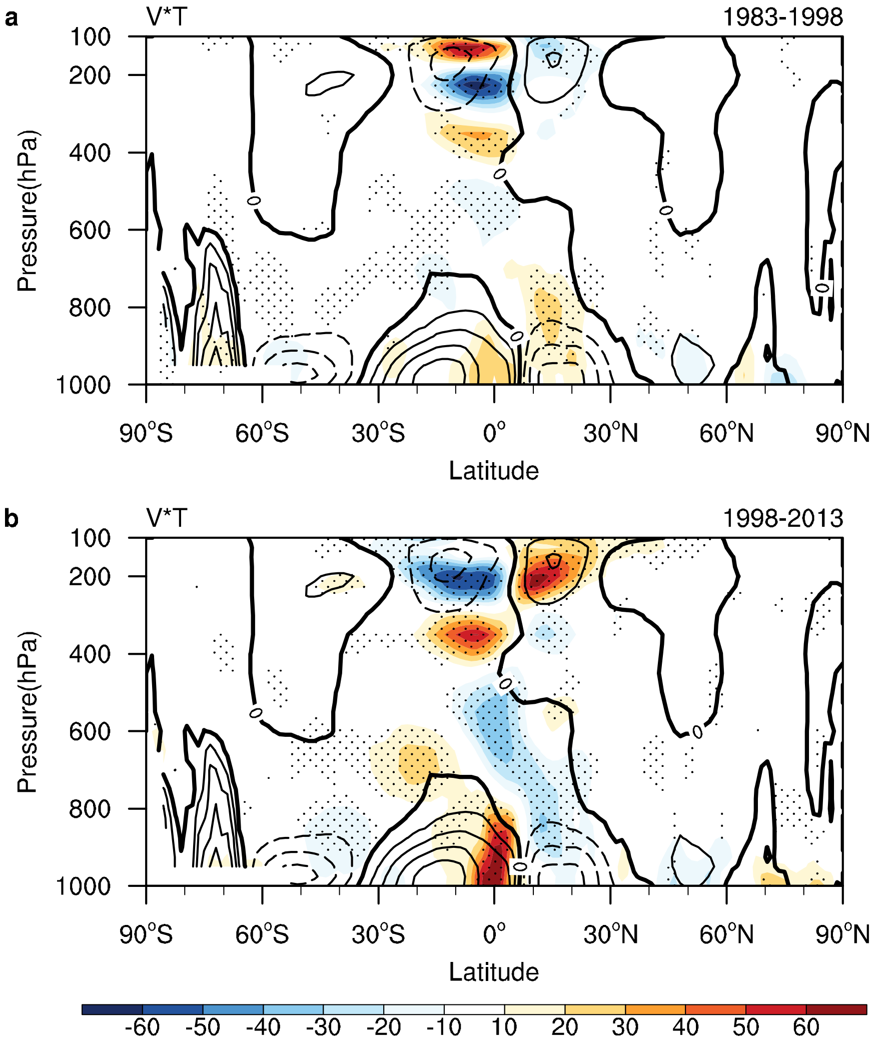

Fig. S8. Climatology and trends of meridional cross section of zonally averaged northward heat flux. Trends of meridional cross section of zonally averaged northward heat flux calculated by multiplying meridional velocity (**V**) and temperature (**T**) for (**a**) the warming period (shading) and (**b**) the hiatus period (shading). Contours are the climatological meridional cross section of zonally averaged northward heat flux. Contour interval is 100 K m s^{-1}. Trends statistically significant at the 5% level based on a Student's *t*-test are dotted.

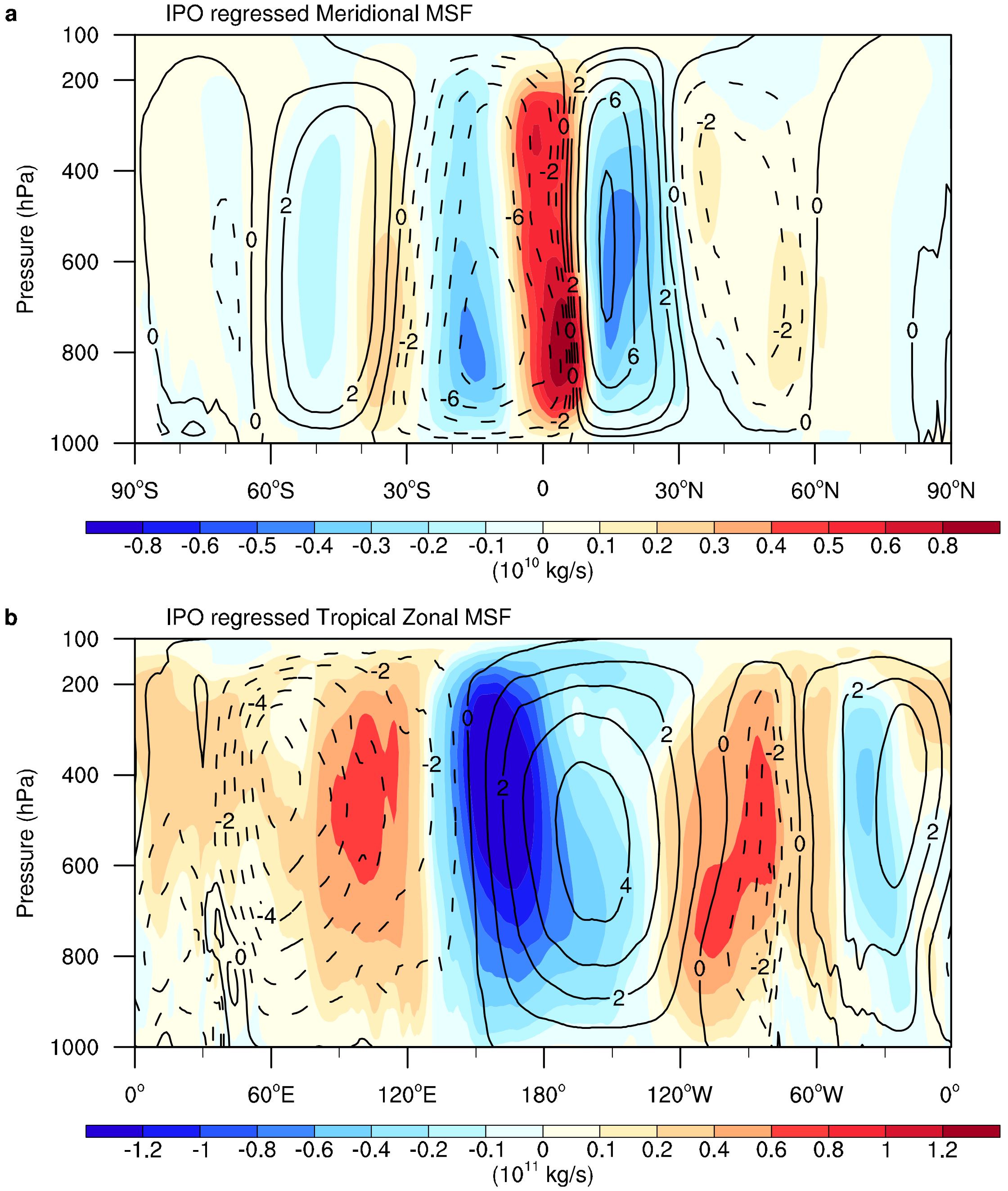

Fig. S9. IPO regressed meridional MSF and tropical zonal MSF. Regression of IPO index[25] on (**a**) meridional MSF and on (**b**) tropical zonal MSF along tropics (5°S-5°N) derived from ERA-20C.

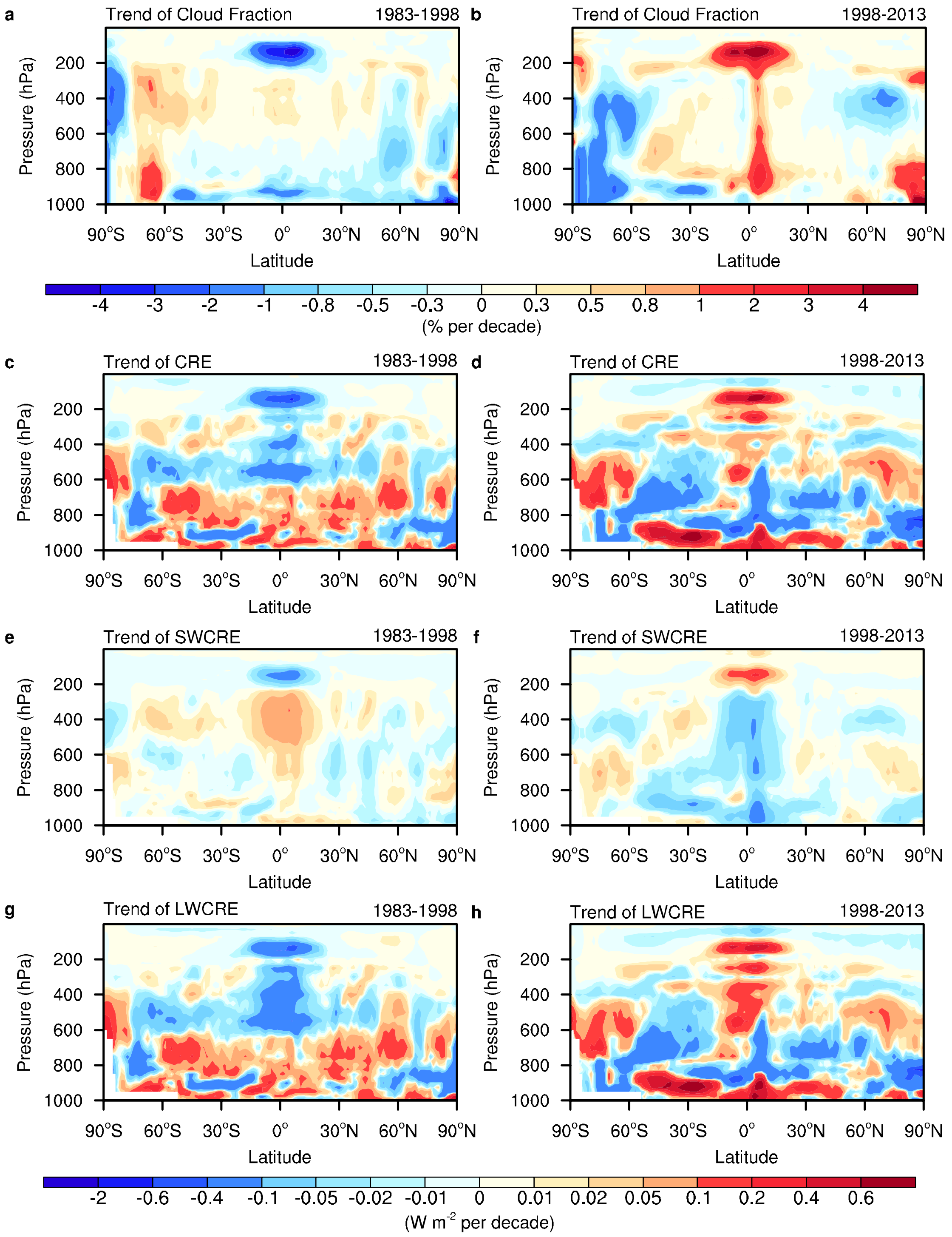

Fig. S10. Trends of cloud fraction and cloud radiative effect (CRE). Trends of zonal mean of annual mean (**a, b**) total cloud fraction and (**c, d**) total CRE as well as (**e, f**) shortwave CRE (SWCRE) and (**g, h**) longwave CRE (LWCRE) for (left) the warming period and (right) the hiatus period.
